# Unrestricted kinematic alignment offers limited functional benefit over mechanical alignment in medial pivot total knee arthroplasty: A randomized controlled trial using conventional instrumentation

**DOI:** 10.1002/ksa.12751

**Published:** 2025-07-18

**Authors:** Amir Koutp, Rene Schroedter, Lukas Leitner, Ines Vielgut, Andreas Leithner, Patrick Sadoghi

**Affiliations:** ^1^ Department of Orthopaedics and Trauma Medical University of Graz Graz Austria; ^2^ Department of Orthopaedics and Trauma Surgery Musculoskeletal University Center Munich (MUM), LMU University Hospital Munich Germany

**Keywords:** CPAK classification, joint awareness, mechanical alignment, patient‐reported outcome measures, randomized controlled trial

## Abstract

**Purpose:**

The aim of this randomized controlled trial was to compare clinical outcomes between kinematic alignment (KA) and mechanical alignment (MA) in total knee arthroplasty (TKA) using a medial‐pivot (MP) prosthesis and conventional instrumentation. The primary hypothesis was that KA would result in improved joint awareness at 2 years postoperatively.

**Methods:**

One hundred patients with end‐stage knee osteoarthritis were enroled between October 2020 and December 2024 and randomized to receive either KA or MA. All surgeries were performed by a single surgeon using the same MP prosthesis. Clinical scores (OKS, WOMAC, KSS, FJS‐12) and radiographic measurements were collected preoperatively and at a 2‐year follow‐up. Subgroup analysis was performed based on coronal knee alignment (Coronal Plane Alignment of the Knee classification).

**Results:**

At 2 years, KA demonstrated statistically significant differences in KSS Pain (*p* = 0.024), WOMAC total (*p* = 0.003) and FJS‐12 (*p* = 0.001) compared to MA. The range of motion did not differ significantly between groups (*p* = 0.201). In subgroup analyses, patients with varus alignment showed a statistically significant and clinically meaningful improvement in WOMAC scores. However, most between‐group differences did not exceed established minimal clinically important difference thresholds.

**Conclusion:**

KA with an MP TKA design was associated with statistically higher functional scores and joint awareness compared to MA, particularly in patients with varus alignment. However, the observed differences were modest, and further studies are warranted to clarify the clinical relevance of KA across phenotypes‐specific subgroups.

**Level of Evidence:**

Level II, randomized controlled trial.

**Abbreviations**
BMI: body mass index
CI: conventional instrumentation
CPAK: Coronal Plane Alignment of the Knee
FJS‐12: Forgotten Joint Score
HKA: hip‐knee‐ankle angle
KA: kinematic alignment
KSS: Knee Society Score
LDFA: lateral distal femoral angle
MA: mechanical alignment
MCID: minimal clinically important difference
MP: medial pivot (prosthesis design)
MPTA: medial proximal tibial angle
OKS: Oxford Knee Score
PS: posterior‐stabilized (prosthesis design)
RCT: randomized controlled trial
ROM: range of motion
SD: standard deviation
TKA: total knee arthroplasty
WOMAC: Western Ontario and McMaster Universities Arthritis Index

## INTRODUCTION

Kinematic alignment (KA) is a resurfacing‐based technique in total knee arthroplasty (TKA) that aims to restore the patient's pre‐arthritic knee anatomy by aligning the prosthetic components with the individual's natural anatomical axes of motion. While not a new approach, KA has gained renewed interest due to its focus on replicating native joint kinematics by respecting the unique geometry and soft tissue balance of each knee [16]. Historically, its wider adoption has been limited by concerns regarding outlier limb alignment, patellar maltracking and potential risk of aseptic loosening. However, these concerns are increasingly being addressed in recent studies, and KA continues to be evaluated as a promising alternative to mechanical alignment (MA) [12, 14, 30].

Several randomized studies have explored the comparison between mechanical and KA, with some reporting clinically and functionally favourable outcomes for KA [5, 8], whereas other studies have found no significant difference in outcomes between the two approaches [10, 33]. While meta‐analyses generally report no significant differences in clinical and functional outcomes between mechanical and KA, they often group various alignment strategies, such as unrestricted, restricted and inverse KA, under a single umbrella term [24, 31, 32]. This methodological limitation, as highlighted by recent systematic appraisals [27], contributes to conflicting results and hinders a clear interpretation of the true efficacy of unrestricted KA. Furthermore, most included studies use cruciate‐retaining or posterior‐stabilized (PS) implant designs and encompass a wide range of alignment phenotypes, which adds further heterogeneity to the pooled analyses. Limited evidence exists regarding the use of medial pivot (MP) designs, which aim to replicate more natural knee kinematics through a medially stabilized ball‐and‐socket mechanism [29]. However, one randomized study employing an MP design with patient‐specific instrumentation (PSI) and restricted KA reported superior patient satisfaction, self‐reported function and joint awareness for KA compared to MA in the early postoperative period [11].

The aim of this study was to compare clinical and functional outcomes of unrestricted KA versus MA TKA using an MP design and conventional instrumentation (CI).

Our hypothesis was that KA would lead to superior joint awareness compared to MA, as measured by the Forgotten Joint Score‐12 (FJS‐12) at 2 years postoperatively, and that secondary differences would be observed in functional outcomes and patient‐reported scores depending on Coronal Plane Alignment of the Knee (CPAK) phenotype.

## METHODS

### Trial design and setting

This monocentric, prospective, randomized controlled, open‐label trial was conducted from October 2020 to December 2024. Ethical approval was obtained from the institutional review board, and the study was prospectively registered at ClinicalTrials.gov (Identifier: NCT04436211).

### Changes to trial protocol

Important amendments to the trial protocol were made prior to data analysis and were approved by the institutional review board. Initially, KA was planned using patient‐specific cutting blocks from computed tomography scans. This technique was later replaced by the use of CI based on Howell's calipered technique [13], which was ultimately used in all patients included in this analysis. The primary outcome was also changed from knee range of motion (ROM), and functional outcomes as Oxford Knee Score (OKS), Western Ontario and McMaster Universities Arthritis Index (WOMAC) and Knee Society Score (KSS) at intervals of 3, 6 and 12 months to the FJS‐12 at 2 years postoperatively. ROM and functional scores remained as secondary outcomes.

### Participants

Patients aged ≥50 years scheduled for unilateral primary TKA for end‐stage knee osteoarthritis (Kellgren–Lawrence grade IV) were eligible. Exclusions included age <50 years, previous fractures of the femur or tibia, septic arthritis, osteotomy, previous partial or total knee arthroplasty, inflammatory arthritis, indication for bilateral TKA and preoperative malalignment exceeding 5° varus or any valgus deformity, as well as clinically relevant joint instability. All patients provided explicit informed consent for study participation.

### Randomization, stratification and allocation concealment

In the broader randomized trial, a total of 433 patients were randomized in a 1:1 ratio to receive either MA or KA total knee arthroplasty. Randomization was conducted using block randomization with randomly varying block sizes of four and six, stratified by age and gender. The sequence was generated using an online platform (www.randomizer.at), and allocation concealment was maintained using sequentially numbered, opaque sealed envelopes handled independently by research personnel uninvolved in patient care.

The current study analyzes a predefined subgroup of patients treated with CI who completed a minimum of 2 years of follow‐up. From the 379 patients treated with CI (171 KA, 208 MA), 50 randomized patients from each group were included in this analysis. No post hoc matching was performed.

### Blinding

The surgeon was unblinded due to the nature of interventions. However, outcome assessors, including clinical examiners and radiographic evaluators, were blinded to group allocation. Patients were also not informed about their alignment group.

### Interventions

All patients underwent implantation of an MP TKA system (GMK Sphere, Medacta). All TKAs in both groups were implanted using antibiotic‐loaded bone cement (PALACOS fast *R* + G, Heraeus). MA was achieved in using a tibia‐first and extension‐gap‐first technique with CIs and an external rotation of the femoral component of 3°. A detailed description was delivered by our study group in previous studies [19, 20]. Unrestricted KA was performed using the calipered technique described by Howell, employing the same extramedullary tibial guide [13].

### Postoperative rehabilitation

Postoperative care followed the standardized rehabilitation protocol as previously described in our prior study [22]. All patients underwent early mobilization on the day of surgery, with structured inpatient physical therapy beginning within 24 h postoperatively. The rehabilitation regimen included ROM exercises, progressive weight‐bearing as tolerated, quadriceps and gait training, and outpatient continuation of therapy after discharge. Progress was monitored through standardized clinical milestones to ensure consistent rehabilitation across both groups.

### Outcome measures

The primary outcome measure was the FJS‐12, assessed 2 years postoperatively. The FJS‐12 evaluates joint awareness during activities of daily living on a scale from 0 to 100; higher scores indicate less joint awareness, reflecting better outcomes [3].

Secondary outcomes included the OKS, WOMAC, KSS and knee ROM, measured by a blinded examiner. The OKS evaluates knee pain and function (scores 0–48, higher scores better) [7], WOMAC measures pain, stiffness and function (total scores 0–96, higher scores worse) [4], and the KSS assesses knee condition and function (scores 0–100, higher scores better) [17].

### Radiographic measurements

All patients underwent skyline view, anterior‐posterior and lateral view of the knee, and long leg X‐rays, preoperatively as part of the preoperative planning. A single experienced observer not involved in the surgical procedures measured the lateral distal femoral angle (LDFA) and the medial proximal tibial angle (MPTA) on these radiographs. LDFA was measured as the lateral angle subtended by a line from the centre of the femoral head to the centre of the intercondylar notch and the other line tangential to the femoral condyles. MPTA was measured as the medial angle subtended by a line from the centre of the tibial spines to the centre of the ankle joint and the other line tangential to the tibial articular surface. Preoperative arithmetic hip‐knee‐ankle angle (aHKA) was calculated by the formula MPTA‐LDFA and JLO was the sum of MPTA and LDFA. Each knee was classified preoperatively according to the CPAK system [25]. While CPAK‐specific reliability was not assessed in this study, excellent inter‐ and intraobserver agreement for frontal plane alignment parameters was previously demonstrated in a prospective analysis from the same cohort, where two observers independently measured pre‐ and postoperative tibial angles with high precision (intraclass correlations > 0.90) [21].

### Sample size calculation

The initial sample size calculation was based on detecting a 5° difference in postoperative knee flexion ROM (SD ± 10°), with *α* = 0.05 and power = 0.80, requiring at least 44 patients per group. To account for attrition, 50 patients per group were enroled. A priori sample size calculation was based on detecting a clinically meaningful difference in knee flexion of 7° with a standard deviation (SD) of 10°, requiring 45 patients per group (*α* = 0.05, power = 0.80). To account for potential dropouts, we included 50 patients per group.

A post hoc power analysis was conducted for the primary outcome FJS‐12. With an observed between‐group difference of 8.4 points and a pooled SD of 11.65, the resulting effect size (Cohen's *d*) was 0.72. Given the final sample size of 50 patients per group, the post hoc power was 94.6%, indicating that the study was adequately powered to detect the observed difference in FJS‐12 between the kinematic and MA groups.

### Statistical analysis

The mean and SD were determined for each measure in each group. Differences in the means of primary outcome measures between groups were analyzed using the non‐parametric Mann–Whitney *U*‐test for non‐normally distributed data, an unpaired *t*‐test for normally distributed data, and chi‐squared tests for categorical data. For each group, we calculated the change from preoperative to 2‐year postoperative scores for OKS, WOMAC and KSS to assess clinical improvement. These change scores were compared between the MA and KA groups. Minimal clinically important differences (MCIDs) were used to interpret the clinical relevance of statistically significant findings. Based on existing literature, MCID thresholds were defined as follows: 4 points for the OKS [2], 10–12 points for the WOMAC [9], 14–16.6 points for the FJS‐12 [6] and 6.3–10.3 points for the KSS [23]. All analyses were performed using statistical software (SPSS Inc. v29, IBM Corp., and JMP v11, SAS). Statistical significance was set at *p* < 0.05.

## RESULTS

### Participant flow and recruitment

From October 2020 to December 2024, a total of 379 patients with end‐stage knee osteoarthritis (Kellgren–Lawrence grade IV) were randomized to receive either KA (*n* = 171) or MA (*n* = 208) using CI. Patients who received PSI were excluded from this analysis.

**Figure 1:**
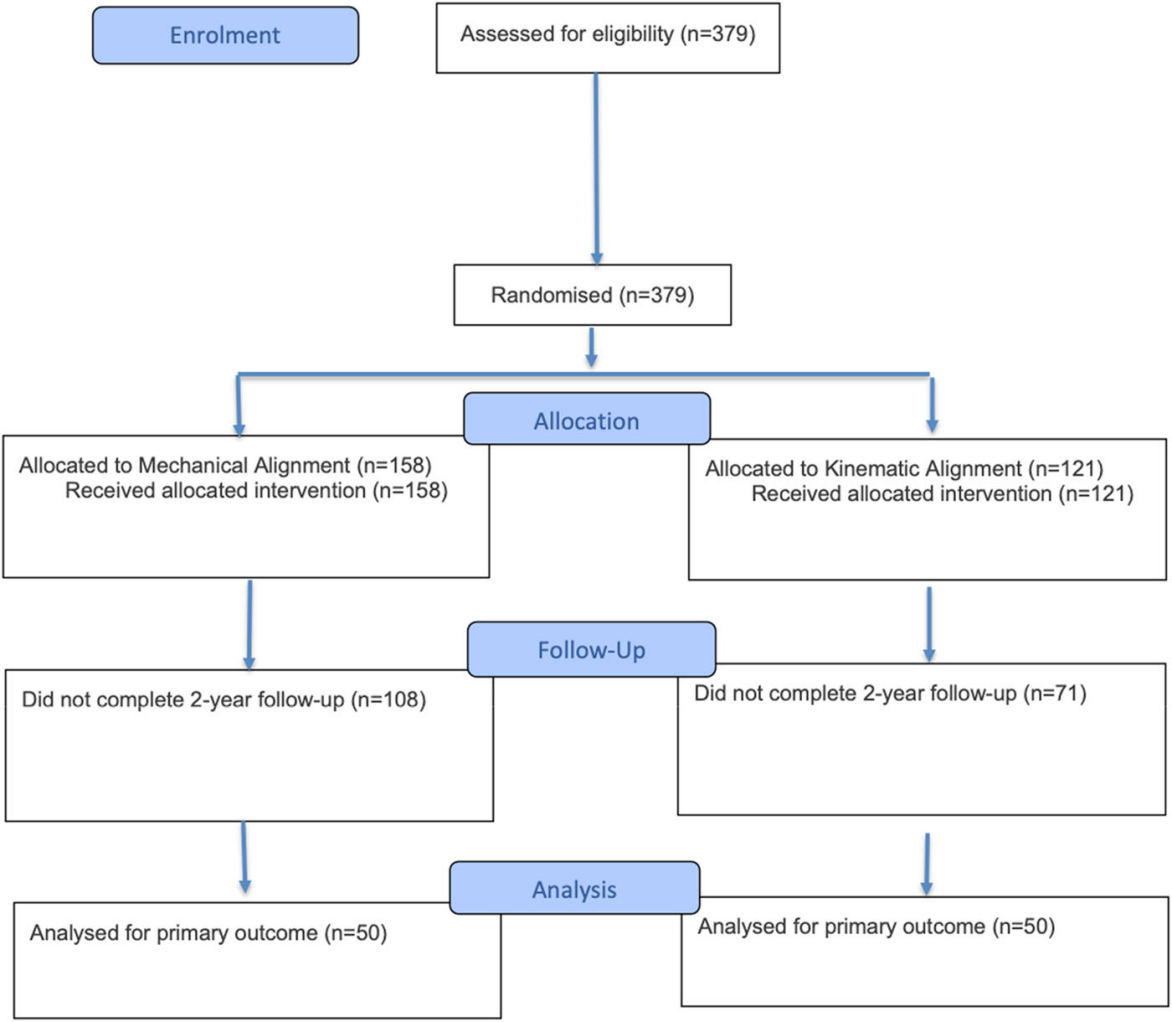
CONSORT flow diagram of patient 208 enrolment, allocation, follow‐up and analysis. A total of 379 patients were assessed for eligibility and randomized to receive either mechanical alignment (MA) or kinematic alignment (KA) total knee arthroplasty. Of the 158 patients allocated to MA, 50 completed the 2‐year follow‐up and were included in the final analysis. Similarly, 50 of the 121 patients in the KA group completed the 2‐year follow‐up and were analyzed for the primary outcome.

After exclusions for insufficient 2‐year follow‐up (KA: *n* = 121; MA: *n* = 158), 50 patients from each group who had undergone surgery as randomized and completed the full 2‐year follow‐up were included in the final intention‐to‐treat analysis. All 100 patients included in the present analysis were randomized and received CI TKA. The full participant flow is illustrated in Figure 1.

### Baseline characteristics

Baseline demographic, radiographic and clinical characteristics were similar between groups. The mean age was 72.52 ± 8.57 years in the KA group and 69.60 ± 9.30 years in the MA group (*p* = 0.106). The proportion of female patients was 58% in the KA group and 60% in the MA group (*p* = 1.000). The body mass index (BMI) was 30.00 ± 4.72 in the KA group and 31.55 ± 6.22 in the MA group (*p* = 0.188). Radiographic alignment values showed no significant group differences: aHKA was −0.60 ± 4.44 in KA versus 0.48 ± 5.40 in MA (*p* = 0.2778); MPTA was 87.44 ± 2.89 versus 88.40 ± 3.85 (*p* = 0.1621); and LDFA was 88.04 ± 2.73 versus 87.92 ± 2.93 (*p* = 0.8326). CPAK phenotype distributions were also comparable (Table 1).

**Table 1:** Baseline demographic and anatomical characteristics of patients in the mechanical alignment (MA) and kinematic alignment (KA) groups.

Variable	MA	KA	*p*‐Value
Age	69.60 ± 9.30	72.52 ± 8.57	0.106
Sex (female, %)	60.0%	58.0%	1.0000
BMI	31.55 ± 6.22	30.00 ± 4.72	0.188
aHKA	0.48 ± 5.40	−0.60 ± 4.44	0.2778
MPTA	88.40 ± 3.85	87.44 ± 2.89	0.1621
LDFA	87.92 ± 2.93	88.04 ± 2.73	0.8326
CPAK			
Varus			
Type I	10 (20%)	13 (26%)	0.634
Type IV	6 (12%)	5 (10%)	1.000
Type VII	0	0	‐
Neutral			
Type II	9 (18%)	11 (22%)	0.797
Type V	7 (14%)	7 (14%)	1.000
Type VIII	0	0	‐
Valgus			
Type III	8 (16%)	7 (14%)	1.000
Type VI	9 (18%)	7 (14%)	0.774
Type IX	1 (2%)	0	1.000

*Note*: Values are presented as mean ± standard deviation or percentage unless otherwise noted. No statistically significant differences were observed between groups across any variable.

Abbreviations: aHKA, arithmetic hip‐knee‐ankle angle; BMI, body mass index; CPAK, Coronal Plane Alignment of the Knee classification; LDFA, lateral distal femoral angle; MPTA, medial proximal tibial angle.

Comparison of preoperative characteristics of the two groups showed no significant differences in age, gender, BMI, alignment parameters, OKS, WOMAC scores or KSS components (pain, function, combined) (Table 2).

**Table 2:** Preoperative clinical scores comparing mechanical alignment (MA) and kinematic alignment (KA) groups.

Parameter	MA (mean ± SD)	KA (mean ± SD)	*p*‐Value	Cohen's *d*
ROM	110.8° ± 21.27°	113.5° ± 14.19°	0.457	0.15
KSS combined preoperative	107.48 ± 28.94	118.44 ± 19.69	0.532	0.06
KSS ROM preoperative	22.56 ± 4.25	23.10 ± 2.84	0.997	0.15
KSS pain preoperative	17.20 ± 10.89	19.60 ± 13.40	0.370	0.20
KSS function preoperative	46.90 ± 22.70	51.00 ± 10.25	0.084	0.23
OKS preoperative	22.88 ± 7.79	22.44 ± 7.62	0.634	−0.06
WOMAC combined preoperative	63.37 ± 13.09	61.85 ± 7.35	0.081	−0.14
WOMAC symptoms preoperative	29.32 ± 8.25	27.00 ± 6.76	0.220	−0.31
WOMAC stiffness preoperative	14.56 ± 3.75	14.08 ± 2.52	0.336	−0.15
WOMAC activity preoperative	108.20 ± 24.6	107.36 ± 13.81	0.184	−0.04

*Note*: Values are presented as mean ± standard deviation. No statistically significant differences were found across any parameter. Cohen's *d* is provided as a measure of effect size.

Abbreviations: KSS, Knee Society Score; OKS, Oxford Knee Score; ROM, range of motion; SD, standard deviation; WOMAC, Western Ontario and McMaster Universities Osteoarthritis Index.

### Numbers analyzed

All 100 randomized patients who met the eligibility criteria and completed the required 2‐year follow‐up were included in the final analysis. There were no protocol deviations, crossovers or exclusions after allocation. The analysis followed an intention‐to‐treat principle.

### Primary outcome

At the 2‐year follow‐up, joint awareness measured by the FJS‐12 was significantly higher in the KA group (65.64 ± 13.28) than in the MA group (57.24 ± 10.01), with a between‐group difference of 8.4 points (*p* = 0.001). Although statistically significant, this difference did not reach the MCID of 16.6 points, suggesting that the improvement in joint perception may not be clinically meaningful.

### Secondary outcomes

At 2 years, the OKS was 42.4 ± 4.3 in the KA group and 40.1 ± 5.7 in the MA group (*p* = 0.048). The total WOMAC score was significantly lower (better) in the KA group (22.82 ± 11.65) compared to the MA group (26.53 ± 10.15, *p* = 0.003). Subscale analysis showed statistical differences in WOMAC symptoms (11.48 ± 5.73 vs. 13.88 ± 5.56, *p* = 0.005) and activity (38.40 ± 20.14 vs. 44.12 ± 18.28, *p* = 0.036), while WOMAC stiffness did not differ significantly (*p* = 0.110). However, none of these differences exceeded the commonly cited MCID thresholds of 10–12 points, indicating they may not be clinically perceptible.

The KSS pain subscore was statistically significantly better in the KA group (47.90 ± 6.23) versus the MA group (45.90 ± 8.12; *p* = 0.024), while the KSS functional score (*p* = 0.081) and combined score (*p* = 0.067) showed non‐significant trends favouring KA.

Knee ROM at 2 years was slightly higher in the KA group (119° ± 10°) compared to the MA group (117° ± 11°), though this was not statistically significant (*p* = 0.201).

At the 1‐year follow‐up, differences between groups in most outcomes were not statistically significant, except for WOMAC symptoms (*p* = 0.015), which again did not exceed MCID.

**Figure 2:**
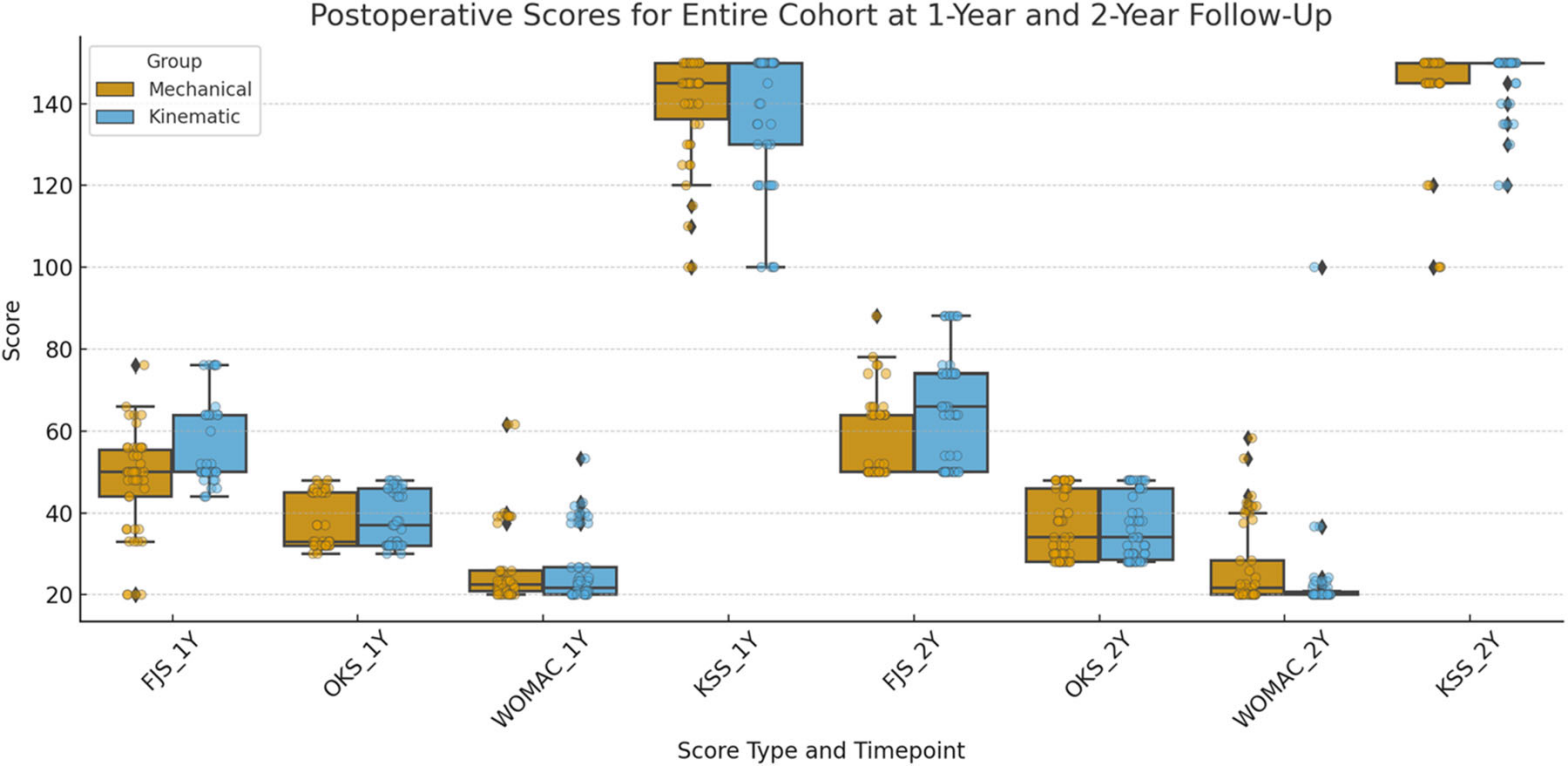
Boxplots of postoperative clinical outcome scores at 1‐ and 2‐year follow‐up for the entire cohort, comparing mechanical alignment (MA) and kinematic alignment (KA) groups. Outcomes include the Forgotten Joint Score (FJS), Oxford Knee Score (OKS), Western Ontario and McMaster Universities Osteoarthritis Index (WOMAC) and the Knee Society Score (KSS). Boxes represent the interquartile range; whiskers extend to 1.5× IQR. Individual data points are overlaid.

A detailed listing of the results of the primary outcome and secondary outcomes is illustrated in Table 3 and Figure 2.

**Table 3:** Postoperative clinical scores at 1‐ and 2‐year follow‐up comparing mechanical alignment (MA) and kinematic alignment (KA) groups.

Parameter	Follow‐up	MA (mean ± SD)	KA (mean ± SD)	*p*‐Value	Cohen's *d*
ROM	Postoperative 2 years	117° ± 11°	119° ± 10°	0.201	0.19
KSS combined	Postoperative 1 year	188.56 ± 13.87	187.76 ± 16.12	0.328	−0.05
	Postoperative 2 years	193.12 ± 13.71	196.38 ± 8.24	0.07	0.29
KSS ROM	Postoperative 1 year	23.56 ± 1.86	23.56 ± 2.02	0.936	0.00
	Postoperative 2 years	23.82 ± 1.87	24.48 ± 1.42	0.078	0.40
KSS pain	Postoperative 1 year	45.60 ± 6.12	44.40 ± 9.18	0.595	−0.15
	Postoperative 2 years	45.90 ± 8.12	47.90 ± 6.23	0.024	0.28
KSS function	Postoperative 1 year	94.40 ± 9,72	94.80 ± 8.14	0.884	0.04
	Postoperative 2 years	98.40 ± 5.48	99.00 ± 3.62	0.930	0.13
OKS	Postoperative 1 year	37.88 ± 6.77	38.52 ± 6.78	0.559	0.09
	Postoperative 2 years	36.64 ± 8.34	36.68 ± 8.11	0.874	0.00
WOMAC combined	Postoperative 1 year	26.22 ± 9.81	26.13 ± 8.59	0.654	−0.01
	Postoperative 2 years	26.53 ± 10.15	22.82 ± 11.65	0.003	−0.34
WOMAC symptoms	Postoperative 1 year	13.80 ± 4.73	11.84 ± 2.55	0.015	−0.52
	Postoperative 2 years	13.88 ± 5.56	11.48 ± 5.73	0.005	−0.43
WOMAC stiffness	Postoperative 1 year	6.48 ± 3.20	6.36 ± 2.27	0.623	−0.04
	Postoperative 2 years	5.68 ± 2.84	4.88 ± 2.53	0.103	−0.04
WOMAC activity	Postoperative 1 year	42.64 ± 17.39	44.52 ± 17.22	0.311	0.11
	Postoperative 2 years	44.12 ± 18.28	38.40 ± 20.14	0.036	−0.30
FJS	Postoperative 1 year	47.56 ± 12.21	55.48 ± 9.70	0.009	0.72
	Postoperative 2 years	57.24 ± 10.01	65.64 ± 13.28	<0.001	0.71

*Note*: Values are presented as mean ± standard deviation (SD). Statistical significance (*p* < 0.05) and effect sizes (Cohen's *d*) are reported to indicate the magnitude and direction of between‐group differences. Positive Cohen's *d* values indicate higher scores in the KA group, and negative values indicate higher scores in the MA group. For the Western Ontario and McMaster Universities Osteoarthritis Index (WOMAC), *lower scores indicate better outcomes* (less pain, stiffness and functional impairment), in contrast to the Knee Society Score (KSS), Oxford Knee Score (OKS) and Forgotten Joint Score (FJS), where higher scores reflect better clinical status. Range of motion (ROM) is reported in degrees.

**Figure 3:**
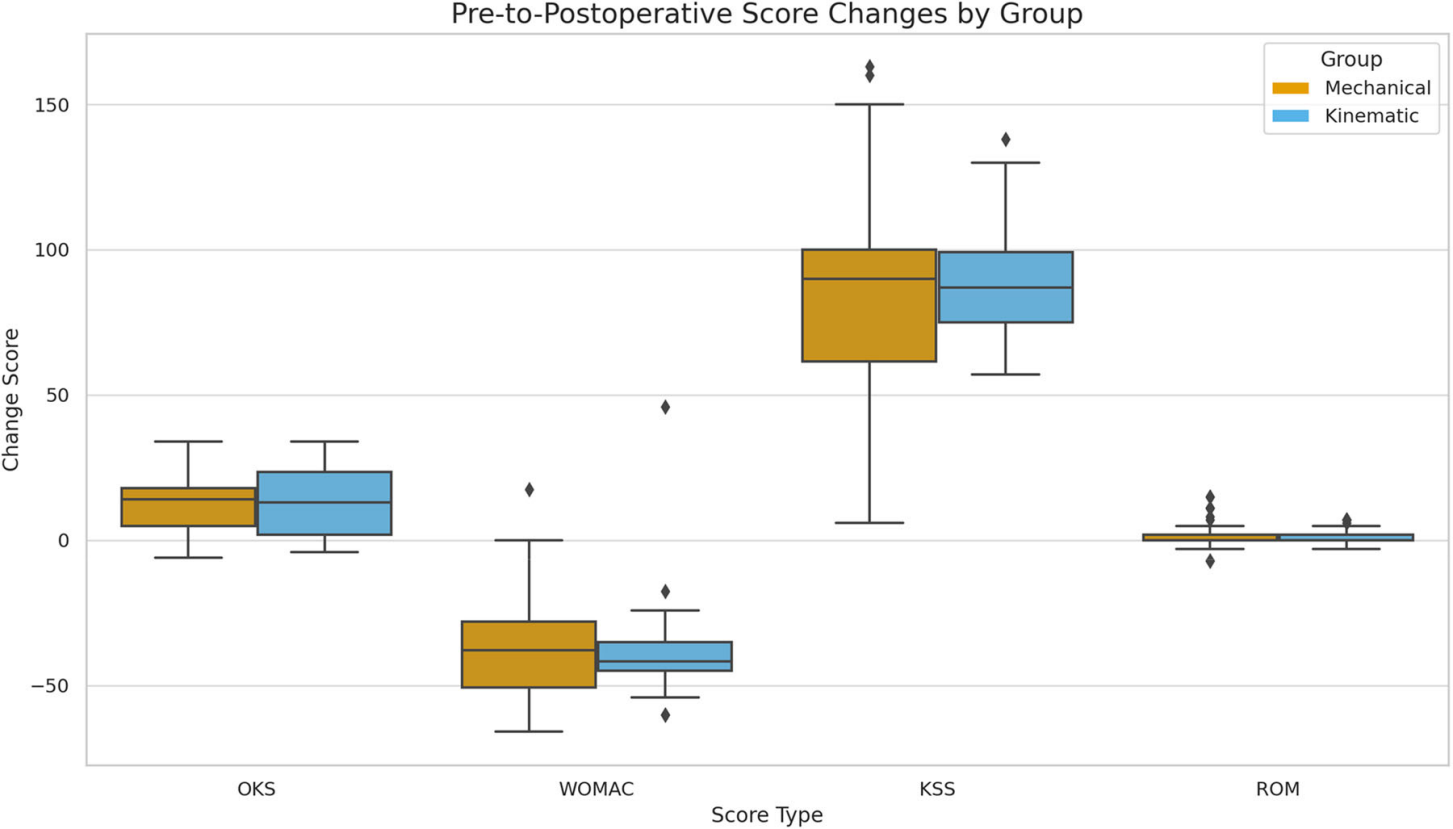
Pre‐to‐postoperative change scores in the Oxford Knee Score (OKS), Western Ontario and McMaster Universities Osteoarthritis Index (WOMAC), Knee Society Score (KSS) and range of motion (ROM) for mechanical alignment (MA) and kinematic alignment (KA) groups. Positive values indicate improvement for OKS, KSS and ROM, while more negative values indicate greater improvement for WOMAC, as lower WOMAC scores reflect better outcomes. Boxplots show the median, interquartile range and outliers (1.5× IQR).

In an additional analysis comparing changes from baseline to 2 years, the mean improvement in combined KSS was 87.42 ± 15.87 in the KA group and 85.64 ± 13.43 in the MA group (*p* = 0.744). The mean improvement in WOMAC was −27.13 ± 15.41 in the KA group and −25.72 ± 14.51 in the MA group (*p* = 0.500), and the OKS improvement was 17.94 ± 8.17 versus 16.56 ± 7.99 (*p* = 0.841), respectively. These differences were not statistically significant. As illustrated in Table 4 and Figure 3.

**Table 4:** Improvement in clinical outcomes from preoperative baseline to 2‐year postoperative follow‐up in the mechanical alignment (MA) and kinematic alignment (KA) groups.

Outcome measure	Follow‐up	MA improvement (mean ± SD)	KA improvement (mean ± SD)	*p*‐Value	Cohen's *d*
Combined KSS	Postoperative 2 years	85.64 ± 13.4	87.42 ± 15.9	0.744	0.12
Combined WOMAC	Postoperative 2 years	−36.83 ± 15.2	−39.03 ± 14.8	0.500	−0.15
OKS	Postoperative 2 years	13.76 ± 8.3	14.24 ± 8.1	0.841	0.06
ROM	Postoperative 2 years	6.2 ± 21.3	5.5 ± 14.2	0.847	−0.04

*Note*: Values are presented as mean ± standard deviation (SD). Cohen's *d* is reported to quantify the effect size of between‐group differences. Positive values indicate greater improvement in the KA group. For the Western Ontario and McMaster Universities Osteoarthritis Index (WOMAC), more negative values reflect greater clinical improvement (as lower WOMAC scores indicate better outcomes). Outcome measures include the Knee Society Score (KSS), Oxford Knee Score (OKS), WOMAC and range of motion (ROM, in degrees).

### Subgroup analysis

Exploratory subgroup analyses were performed based on CPAK phenotypes, grouped into varus (Types I, IV, VII), neutral (Types II, V, VIII) and valgus (Types III, VI, IX) morphotypes.

**Figure 4:**
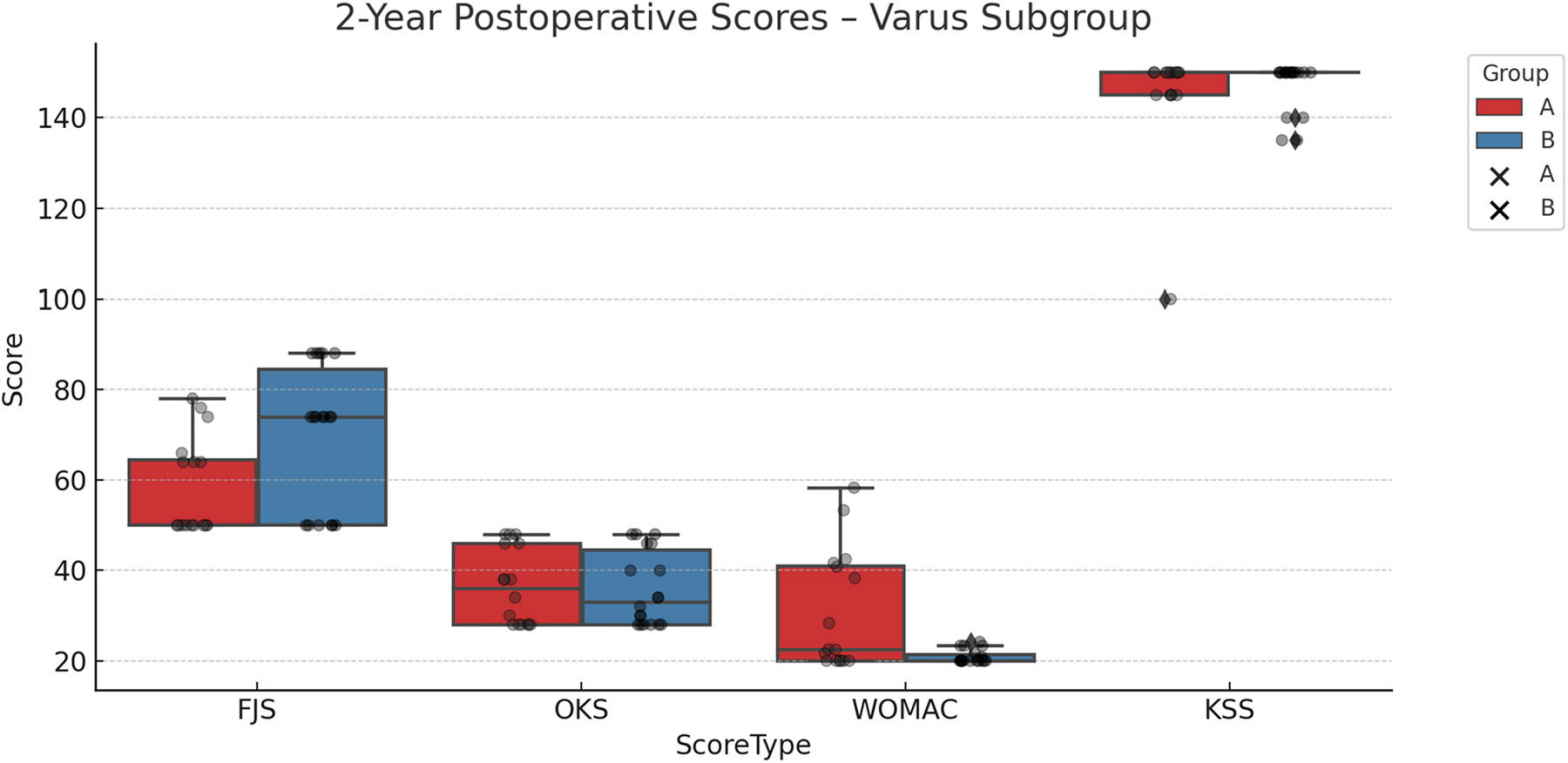
Two‐year postoperative outcome scores for the varus phenotype subgroup, comparing mechanical alignment (MA, red) and kinematic alignment (KA, blue) groups. Boxplots display score distributions for the Forgotten Joint Score (FJS), Oxford Knee Score (OKS), Western Ontario and McMaster Universities Osteoarthritis Index (WOMAC) and Knee Society Score (KSS). Higher scores indicate better outcomes for FJS, OKS and KSS, while lower scores indicate better outcomes for WOMAC. Individual data points are overlaid to show distribution and variation within each group.

Among patients with varus phenotypes, those in the KA group demonstrated significantly better outcomes in the total WOMAC score at 2 years (20.92 ± 1.5 vs. 30.63 ± 13.12 in the MA group, *p* = 0.039). Additionally, FJS‐12 scores favoured KA both at 1 year (60.33 ± 12.12 vs. 49.63 ± 14.09, *p* = 0.017) and 2 years (69.89 ± 15.59 vs. 58.50 ± 10.72, *p* = 0.046). However, the observed FJS‐12 difference of 11.4 points, while statistically significant, did not exceed the established MCID of 16.6, suggesting limited clinical relevance. The results are illustrated in Table 5 and Figure 4.

**Table 5:** Postoperative clinical scores at 1‐ and 2‐year follow‐up in patients with a varus phenotype, comparing mechanical alignment (MA) and kinematic alignment (KA).

		Varus phenotype			
Parameter	Follow‐up	MA (mean ± SD)	KA (mean ± SD)	*p*‐Value	Cohen's *d*
KSS	Postoperative 1 year	177.44 ± 15.21	186.89 ± 17.53	0.055	0.57
	Postoperative 2 years	193.25 ± 13.91	197.00 ± 5.90	0.237	0.36
OKS	Postoperative 1 year	39.25 ± 6.73	40.89 ± 7.01	0.365	0.24
	Postoperative 2 years	36.38 ± 8.43	35.78 ± 8.20	0.959	−0.07
WOMAC	Postoperative 1 year	26.56 ± 11.17	28.52 ± 10.32	0.574	0.18
	Postoperative 2 years	30.63 ± 13.12	20.92 ± 1.51	0.039	−1.07
FJS	Postoperative 1 year	49.63 ± 14.09	60.33 ± 12.12	0.017	0.82
	Postoperative 2 years	58.50 ± 10.72	69.89 ± 15.59	0.046	0.84

*Note*: Values are presented as mean ± standard deviation (SD). Effect sizes are reported as Cohen's *d*, with positive values indicating better outcomes in the KA group and negative values indicating better outcomes in the MA group. For the Western Ontario and McMaster Universities Osteoarthritis Index (WOMAC), lower scores represent better outcomes (i.e., less pain and functional limitation). Higher scores reflect better outcomes for the Knee Society Score (KSS), Oxford Knee Score (OKS) and Forgotten Joint Score (FJS).

**Figure 5:**
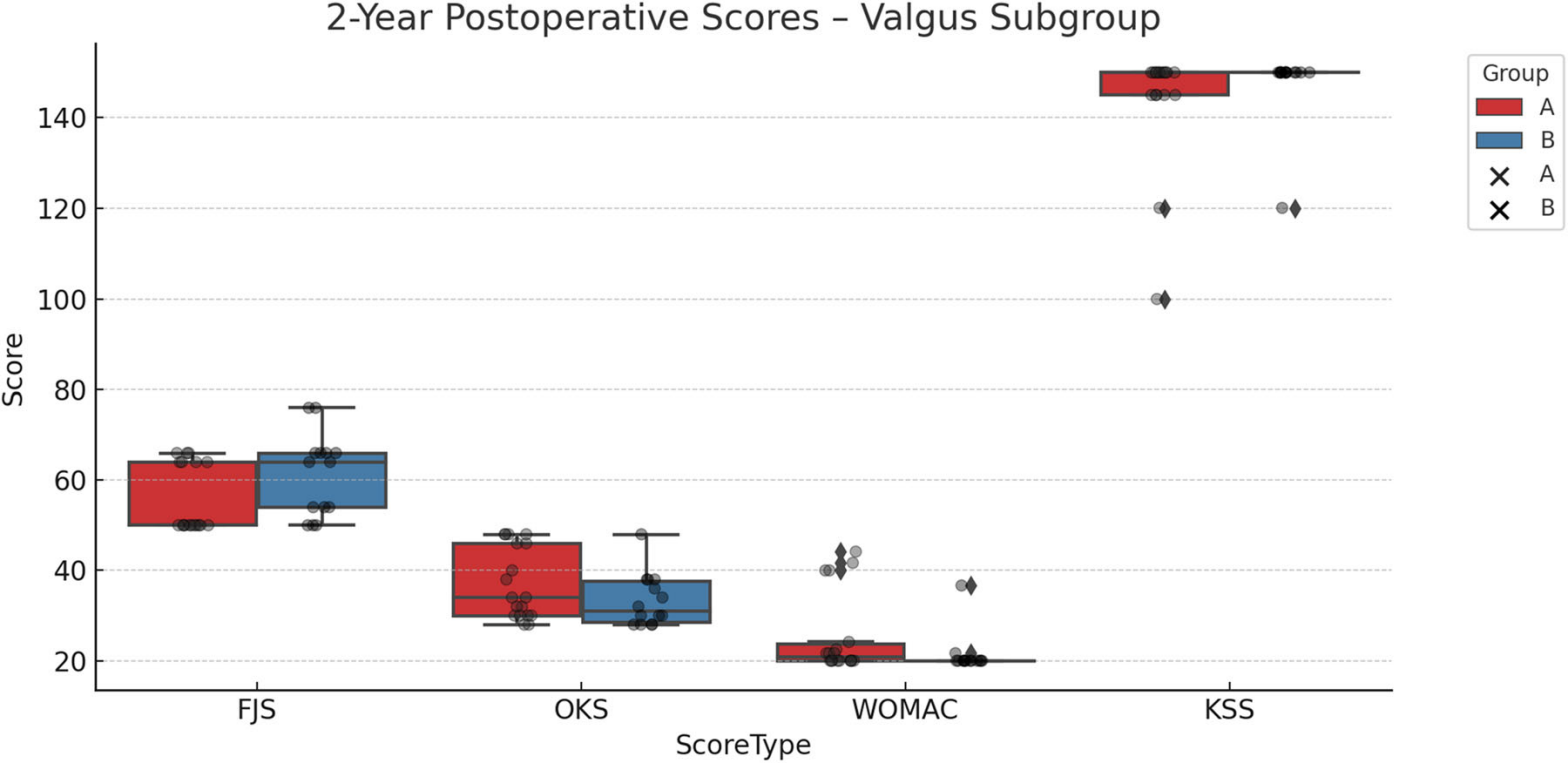
Two‐year postoperative outcome scores for the valgus phenotype subgroup, comparing mechanical alignment (MA, red) and kinematic alignment (KA, blue) groups. Boxplots represent group distributions for the Forgotten Joint Score (FJS), Oxford Knee Score (OKS), Western Ontario and McMaster Universities Osteoarthritis Index (WOMAC) and Knee Society Score (KSS). Higher scores reflect better outcomes for FJS, OKS and KSS, whereas lower scores indicate better outcomes for WOMAC. Data points are overlaid to visualize individual variation within each group.

In patients with valgus phenotypes, KA was also associated with a statistically significantly higher FJS‐12 score at 2 years (61.14 ± 9.11 vs. 55.78 ± 7.48, *p* = 0.049). As with the varus group, the difference did not surpass the MCID threshold, and no other clinical outcomes showed significant differences between alignment strategies in this subgroup. The results are illustrated in Table 6 and Figure 5.

**Table 6:** Postoperative clinical scores at 1‐ and 2‐year follow‐up in patients with a valgus phenotype, comparing mechanical alignment (MA) and kinematic alignment (KA) groups.

		Valgus phenotype			
Parameter	Follow‐up	MA (mean ± SD)	KA (mean ± SD)	*p*‐Value	Cohen's *d*
KSS	Postoperative 1 year	192.50 ± 12.25	189.36 ± 15.44	0.613	−0.22
	Postoperative 2 years	193.11 ± 14.38	197.64 ± 8.82	0.054	0.39
OKS	Postoperative 1 year	37.17 ± 6.68	37.29 ± 6.81	1.000	0.02
	Postoperative 2 years	37.22 ± 7.98	33.29 ± 5.79	0.168	−0.57
WOMAC	Postoperative 1 year	28.10 ± 11.22	24.94 ± 8.13	0.135	−0.33
	Postoperative 2 years	25.42 ± 8.94	21.30 ± 4.44	0.071	−0.60
FJS	Postoperative 1 year	45.33 ± 12.50	51.71 ± 6.79	0.377	0.65
	Postoperative 2 years	55.78 ± 7.48	61.14 ± 9.11	0.049	0.64

*Note*: Values are presented as mean ± standard deviation (SD). Cohen's *d* effect sizes are included to quantify the magnitude and direction of group differences, with positive values indicating better outcomes in the KA group and negative values indicating better outcomes in the MA group. Note that for the Western Ontario and McMaster Universities Osteoarthritis Index (WOMAC), lower scores indicate better clinical status, whereas higher scores denote improvement for the Knee Society Score (KSS), Oxford Knee Score (OKS) and Forgotten Joint Score (FJS).

**Figure 6:**
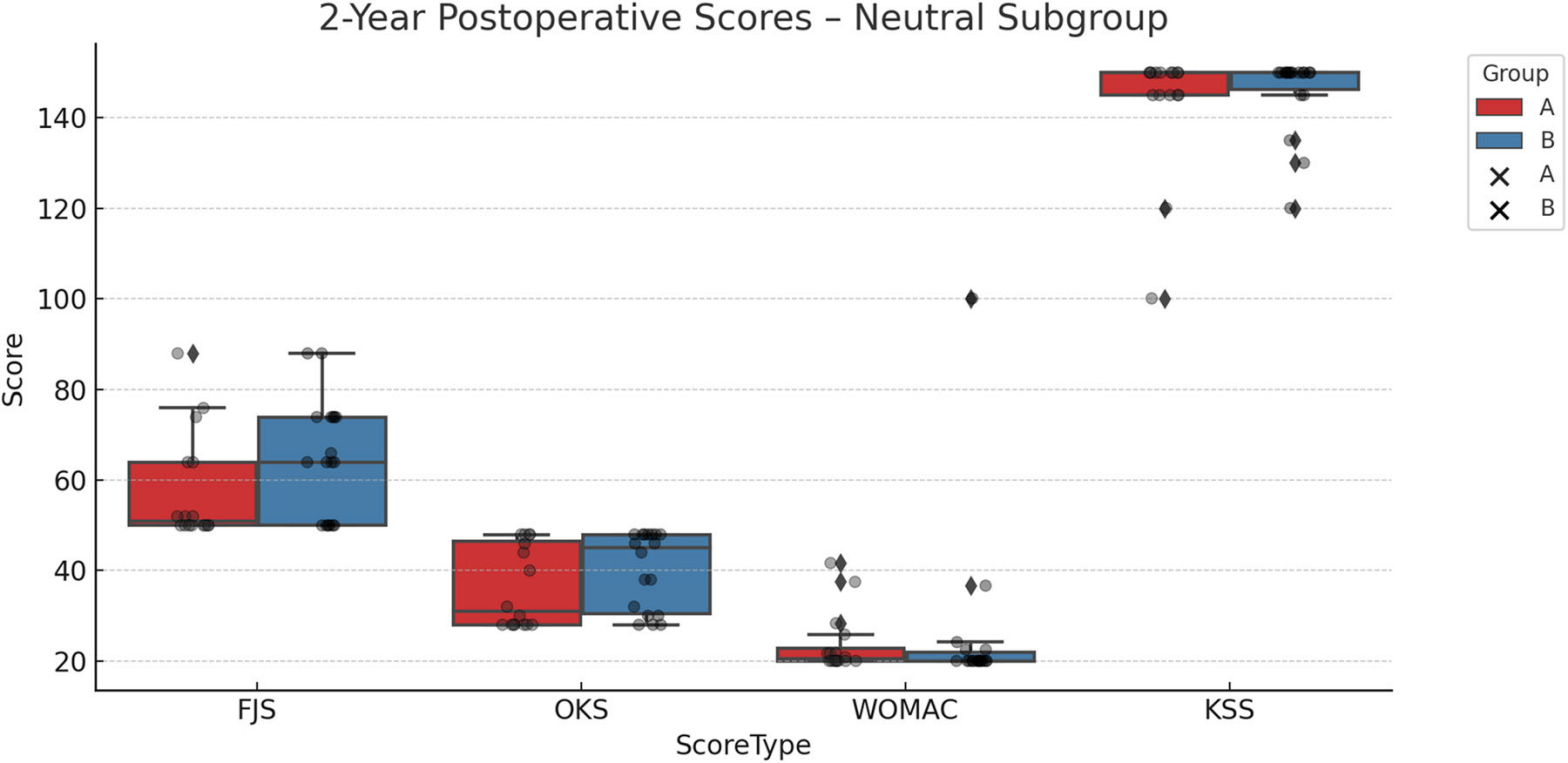
Two‐year postoperative outcome scores for the neutral phenotype subgroup, comparing mechanical alignment (MA, red) and kinematic alignment (KA, blue) groups. Boxplots show the distribution of scores for the Forgotten Joint Score (FJS), Oxford Knee Score (OKS), Western Ontario and McMaster Universities Osteoarthritis Index (WOMAC) and Knee Society Score (KSS). Higher scores indicate better outcomes for FJS, OKS and KSS. In contrast, lower scores reflect better outcomes for WOMAC. Individual patient scores are overlaid as scatter points to illustrate variation within each group.

For patients with neutral phenotypes, no statistically significant differences were observed between KA and MA in any of the outcome measures at either follow‐up timepoint, as illustrated in Table 7 and Figure 6.

**Table 7:** Postoperative clinical scores at 1‐ and 2‐year follow‐up in patients with a neutral phenotype, comparing mechanical alignment (MA) and kinematic alignment (KA) groups.

		Neutral phenotype			
Parameter	Follow‐up	MA (mean ± SD)	KA (mean ± SD)	*p*‐Value	Cohen's *d*
KSS	Postoperative 1 year	195.25 ± 5.40	187.39 ± 16.01	0.403	−0.64
	Postoperative 2 years	193.00 ± 13.64	194.78 ± 9.85	0.347	0.15
OKS	Postoperative 1 year	37.31 ± 7.13	37.11 ± 6.20	0.851	−0.03
	Postoperative 2 years	36.25 ± 9.14	40.22 ± 8.54	0.175	0.45
WOMAC	Postoperative 1 year	23.75 ± 6.00	24.67 ± 6.79	0.825	0.14
	Postoperative 2 years	23.70 ± 6.68	25.88 ± 18.92	0.384	0.15
FJS	Postoperative 1 year	48.00 ± 10.03	53.56 ± 6.81	0.251	0.66
	Postoperative 2 years	57.63 ± 12.03	64.89 ± 12.87	0.175	0.58

*Note*: Values are reported as mean ± standard deviation (SD). Cohen's *d* effect sizes quantify the magnitude and direction of between‐group differences, where positive values indicate better outcomes in the KA group and negative values indicate better outcomes in the MA group. For the Western Ontario and McMaster Universities Osteoarthritis Index (WOMAC), lower scores reflect better outcomes, whereas higher scores reflect better clinical status for the Knee Society Score (KSS), Oxford Knee Score (OKS) and Forgotten Joint Score (FJS).

These subgroup analyses suggest a possible trend toward improved joint perception and function in varus and valgus morphotypes following KA. However, none of the observed between‐group differences met the threshold for minimal clinical importance.

### Harms and adverse events

Two patients in the KA group experienced complications requiring revision surgery. One case involved an acute postoperative infection, and the other resulted from a traumatic patellar dislocation. No complications or revisions were reported in the MA group. No intraoperative complications were observed in either group.

## DISCUSSION

This randomized controlled trial compared the clinical outcomes of KA and MA in total knee TKA using an MP prosthesis and CI. The primary hypothesis was that KA would improve joint awareness, measured by the FJS‐12, and enhance functional outcomes, with variation based on coronal alignment phenotype defined by the CPAK classification.

At the 2‐year follow‐up, the KA group showed higher FJS‐12 scores compared to the MA group; however, the mean difference of 8.4 points did not exceed the established MCID of 16.6. Thus, while the trend favoured KA, the clinical relevance remains uncertain. Similar modest or non‐significant differences have been reported by Shelton et al. [28] and Young et al. [33], reflecting ongoing variability in the literature regarding the impact of alignment strategy on joint awareness.

We also assessed improvements from baseline to 2 years in secondary outcome measures. Although KA showed greater mean improvements in the KSS, OKS and WOMAC, none of these changes reached statistical significance, and most did not surpass MCID thresholds. These results suggest that the alignment strategy may have limited overall influence on perceived recovery in the general cohort.

Subgroup analysis according to CPAK phenotype suggested that patients with varus alignment may benefit more from KA, particularly in WOMAC scores; however, it did not exceed the MCID threshold of 10–12 points in this group. FJS‐12 improvements in the varus subgroup fell short of the MCID. These observations may indicate phenotype‐specific benefits of KA, though subgroup analyses were exploratory and underpowered.

The influence of prosthetic design should also be considered. The MP design is intended to reproduce native knee kinematics, particularly in the medial compartment. Bauer et al. demonstrated in cadaveric studies that KA with MP TKA restored more physiologic motion than MA [1]. However, meta‐analyses by Kakoulidis et al. and van Essen et al. found no consistent superiority of MP implants or KA over conventional techniques in terms of satisfaction or outcomes, underscoring the need for a nuanced view of alignment‐implant interactions [10, 18].

In terms of complications, two revisions occurred in the KA group (one for infection, one for traumatic patellar dislocation), neither of which was related to alignment. No revisions were required in the MA group. While reassuring, the low number of events precludes definitive conclusions about safety or durability.

These findings are consistent with previous studies reporting low and comparable revision rates between mechanical and KA techniques [refs]. For example, a systematic review by Roussot et al. [26] found no significant difference in revision risk between the two alignment philosophies. Similarly, Howell et al. [15] reported a 6‐year implant survival rate of over 97% for KA without an increased risk of complications. Our results support the growing body of evidence suggesting that KA does not increase early postoperative risk, although larger and longer‐term studies are needed to confirm long‐term safety.

### Clinical implications

Our findings suggest that while routine use of KA cannot be broadly recommended based on the current evidence, there may be potential benefits in selected phenotypes, particularly varus‐aligned patients. These preliminary findings may inform individualized surgical planning but require confirmation in larger trials.

### Limitations

This study has several limitations. First, the sample size was adequate for the primary outcome but limited for subgroup comparisons. Second, radiographic assessments were performed by a single observer, although previous data from this cohort showed high inter‐ and intraobserver reliability. Third, domains related to patient expectations and satisfaction (KSS subscales) were not recorded. Fourth, only a single implant type (MP design) was evaluated, which limits generalizability. Fifth, although operative time was documented, it was not included in the original outcomes and thus not analyzed. Finally, a notable proportion of patients lacked complete 2‐year follow‐up due to the timing of enrolment relative to the database lock. This may introduce a degree of attrition bias despite an intention‐to‐treat design. While the original protocol specified a minimum follow‐up of 12 months, the primary outcome was later revised to the FJS at 24 months to better reflect long‐term joint awareness. This amendment was made before data analysis and was approved by the institutional review board.

## CONCLUSION

KA using an MP TKA design was associated with statistically significant improvements in selected outcomes compared to MA, though most differences did not reach MCID thresholds. In varus‐aligned patients, WOMAC improvements approached MCID, suggesting possible phenotype‐specific benefits. These findings emphasize the need for further high‐quality RCTs to clarify the role of alignment.

## AUTHOR CONTRIBUTIONS

*Study design, data collection, statistical analysis, manuscript writing*: Amir Koutp. *Data analysis, statistical consultation, manuscript editing*: Lukas Leitner. *Data acquisition, radiographic analysis, manuscript editing*: Rene Schroedter. *Data analysis, interpretation, manuscript review*: Ines Vielgut. *Conceptualization, critical manuscript revision*: Andreas Leithner. *Principal investigator, study conception, supervision, final manuscript approval*: Patrick Sadoghi.

## CONFLICTS OF INTEREST STATEMENT

Andreas Leithner received industry grants from DePuy Synthes, Johnson & Johnson, alphamed and Medacta. Patrick Sadoghi received Industry grants from DePuy Synthes, Johnson & Johnson, alphamed and Medacta; Editorial Board Member for JOA, KSSTA and Arthroscopy. The remaining authors declare no conflicts of interest.

## ETHICS STATEMENT

This study was approved by the Ethics Committee of the Medical University of Graz (31‐176 ex 18/19). All patients provided informed consent.

## Data Availability

Data are available from the corresponding author upon reasonable request.

## References

[1] Bauer L , Woiczinski M , Thorwächter C , Müller PE , Holzapfel BM , Niethammer TR , et al. Influence of kinematic alignment on femorotibial kinematics in medial stabilized TKA design compared to mechanical alignment. Arch Orthop Trauma Surg. 2022;143(7):4339–4347. 36282314 10.1007/s00402-022-04661-5 PMC10293425

[2] Beard DJ , Harris K , Dawson J , Doll H , Murray DW , Carr AJ , et al. Meaningful changes for the Oxford hip and knee scores after joint replacement surgery. J Clin Epidemiol. 2015;68(1):73–79. 25441700 10.1016/j.jclinepi.2014.08.009 PMC4270450

[3] Behrend H , Giesinger K , Giesinger JM , Kuster MS . The “Forgotten Joint” as the ultimate goal in joint arthroplasty. J Arthroplasty. 2012;27(3):430–436.e1. 22000572 10.1016/j.arth.2011.06.035

[4] Bellamy N , Buchanan WW , Goldsmith CH , Campbell J , Stitt LW . Validation study of WOMAC: a health status instrument for measuring clinically important patient relevant outcomes to antirheumatic drug therapy in patients with osteoarthritis of the hip or knee. J Rheumatol. 1988;15(12):1833–1840. 3068365

[5] Calliess T , Bauer K , Stukenborg‐Colsman C , Windhagen H , Budde S , Ettinger M . PSI kinematic versus non‐PSI mechanical alignment in total knee arthroplasty: a prospective, randomized study. Knee Surg Sports Traumatol Arthrosc. 2017;25(6):1743–1748. 27120192 10.1007/s00167-016-4136-8

[6] Clement ND , Scott CEH , Hamilton DF , MacDonald D , Howie CR . Meaningful values in the Forgotten Joint Score after total knee arthroplasty. Bone Jt J. 2021;103–B(5):846–854. 10.1302/0301-620X.103B5.BJJ-2020-0396.R1 33934639

[7] Dawson J , Fitzpatrick R , Murray D , Carr A . Questionnaire on the perceptions of patients about total knee replacement. J Bone Jt Surg Br. 1998;80(1):63–69. 10.1302/0301-620x.80b1.7859 9460955

[8] Dossett HG , Deckey DG , Clarke HD , Spangehl MJ . Individualizing a total knee arthroplasty with three‐dimensional planning. J Am Acad Orthop Surg Glob Res Rev. 2024;8(3):e24.00023. 10.5435/JAAOSGlobal-D-24-00023 PMC10923344 38478756

[9] Escobar A , Quintana JM , Bilbao A , Aróstegui I , Lafuente I , Vidaurreta I . Responsiveness and clinically important differences for the WOMAC and SF‐36 after total knee replacement. Osteoarthr Cartil. 2007;15(3):273–280. 10.1016/j.joca.2006.09.001 17052924

[10] Van Essen J , Stevens J , Dowsey MM , Choong PF , Babazadeh S . Kinematic alignment results in clinically similar outcomes to mechanical alignment: systematic review and meta‐analysis. Knee. 2023;40:24–41. 36403396 10.1016/j.knee.2022.11.001

[11] Ettinger M , Tuecking LR , Savov P , Windhagen H . Higher satisfaction and function scores in restricted kinematic alignment versus mechanical alignment with medial pivot design total knee arthroplasty: a prospective randomised controlled trial. Knee Surg Sports Traumatol Arthrosc. 2024;32(5):1275–1286. 38501253 10.1002/ksa.12143

[12] van de Graaf VA , Shen TS , Wood JA , Chen DB , MacDessi SJ . Addressing sagittal plane imbalance in primary total knee arthroplasty. Bone Jt Open. 2024;5(8):681–687. 39155644 10.1302/2633-1462.58.BJO-2024-0040.R1 PMC11331267

[13] Howell SM . Calipered kinematically aligned total knee arthroplasty: an accurate technique that improves patient outcomes and implant survival. Orthopedics. 2019;42(3):126–135. 31099877 10.3928/01477447-20190424-02

[14] Howell SM , Papadopoulos S , Kuznik K , Ghaly LR , Hull ML . Does varus alignment adversely affect implant survival and function six years after kinematically aligned total knee arthroplasty? Int Orthop. 2015;39(11):2117–2124. 25823516 10.1007/s00264-015-2743-5

[15] Howell SM , Shelton TJ , Hull ML . Implant survival and function ten years after kinematically aligned total knee arthroplasty. J Arthroplasty. 2018;33(12):3678–3684. 30122435 10.1016/j.arth.2018.07.020

[16] Howell SM , Kuznik K , Hull ML , Siston RA . Results of an initial experience with custom‐fit positioning total knee arthroplasty in a series of 48 patients. Orthopedics. 2008;31(9):857–863. 18814593 10.3928/01477447-20080901-15

[17] Insall JN , Dorr LD , Scott RD , Scott WN . Rationale of the Knee Society clinical rating system. Clin Orthop Relat Res. 1989;248:13–14. 2805470

[18] Kakoulidis P , Panagiotidou S , Profitiliotis G , Papavasiliou K , Tsiridis E , Topalis C . Medial pivot design does not yield superior results compared to posterior‐stabilised total knee arthroplasty: a systematic review and meta‐analysis of randomised control trials. Knee Surg Sports Traumatol Arthrosc. 2023;31(9):3684–3700. 36522493 10.1007/s00167-022-07238-2

[19] Kastner N , Gruber G , Aigner BA , Friesenbichler J , Pechmann M , Fürst F , et al. Sex‐related outcome differences after implantation of low‐contact‐stress mobile‐bearing total knee arthroplasty. Int Orthop. 2012;36(7):1393–1397. 22270864 10.1007/s00264-012-1486-9 PMC3385906

[20] Kastner N , Sternbauer S , Friesenbichler J , Vielgut I , Wolf M , Glehr M , et al. Impact of the tibial slope on range of motion after low‐contact‐stress, mobile‐bearing, total knee arthroplasty. Int Orthop. 2014;38(2):291–295. 24346515 10.1007/s00264-013-2242-5 PMC3923942

[21] Koutp A , Clar C , Leitner L , Fischerauer S , Reinbacher P , Leithner A , et al. Accuracy of conventional instrumentation is dependent on alignment philosophy using the identical surgical technique in total knee arthroplasty. J Knee Surg. 2024;37(01):020–025. 10.1055/a-2176-4767 37714215

[22] Koutp A , Hauer G , Leitner L , Kaltenegger L , Fischerauer S , Clar C , et al. Less induction time and postoperative pain using spinal anesthesia versus general anesthesia with or without the use of peripheral nerve blocks in total knee arthroplasty. J Arthroplasty. 2024;39(4):904–909. 37852447 10.1016/j.arth.2023.10.018

[23] Lizaur‐Utrilla A , Gonzalez‐Parreño S , Martinez‐Mendez D , Miralles‐Muñoz FA , Lopez‐Prats FA . Minimal clinically important differences and substantial clinical benefits for Knee Society Scores. Knee Surg Sports Traumatol Arthrosc. 2020;28(5):1473–1478. 31111184 10.1007/s00167-019-05543-x

[24] Luo Z , Zhou K , Peng L , Shang Q , Pei F , Zhou Z . Similar results with kinematic and mechanical alignment applied in total knee arthroplasty. Knee Surg Sports Traumatol Arthrosc. 2020;28(6):1720–1735. 31250055 10.1007/s00167-019-05584-2

[25] MacDessi SJ , Griffiths‐Jones W , Harris IA , Bellemans J , Chen DB . Coronal Plane Alignment of the Knee (CPAK) classification: a new system for describing knee phenotypes. Bone Jt J. 2021;103–B(2):329–337. 10.1302/0301-620X.103B2.BJJ-2020-1050.R1 PMC7954147 33517740

[26] Roussot MA , Vles GF , Oussedik S . Clinical outcomes of kinematic alignment versus mechanical alignment in total knee arthroplasty: a systematic review. EFORT Open Rev. 2020;5(8):486–497. 32953134 10.1302/2058-5241.5.190093 PMC7484715

[27] Saffarini M , Canetti R , Henry J , Michalewska K , Müller JH , Hirschmann MT . Sparse and inconsistent reporting of pre‐ and post‐operative radiographic angles of total knee arthroplasty using true unrestricted kinematic alignment: an umbrella review and secondary meta‐analysis. Knee Surg Sports Traumatol Arthrosc. 2025;33(3):997–1014. 39460622 10.1002/ksa.12494

[28] Shelton TJ , Gill M , Athwal G , Howell SM , Hull ML . Outcomes in patients with a calipered kinematically aligned TKA that already had a contralateral mechanically aligned TKA. J Knee Surg. 2021;34(1):87–093. 31288274 10.1055/s-0039-1693000

[29] Shimmin A , Martinez‐Martos S , Owens J , Iorgulescu AD , Banks S . Fluoroscopic motion study confirming the stability of a medial pivot design total knee arthroplasty. Knee. 2015;22(6):522–526. 25999125 10.1016/j.knee.2014.11.011

[30] Sun B , Xu Y , Wang G , Chen L , Luo F , Yu G , et al. Comparison of patellar tracking following kinematic alignment versus mechanical alignment total knee arthroplasty via the mini‐subvastus approach. Orthop Surg. 2025;17:1369–1377. 40059658 10.1111/os.70016 PMC12050176

[31] Tian G , Wang L , Liu L , Zhang Y , Zuo L , Li J . Kinematic alignment versus mechanical alignment in total knee arthroplasty: an up‐to‐date meta‐analysis. J Orthop Surg. 2022;30(3):10225536221125952. 10.1177/10225536221125952 36250421

[32] Woon JTK , Zeng ISL , Calliess T , Windhagen H , Ettinger M , Waterson HB , et al. Outcome of kinematic alignment using patient‐specific instrumentation versus mechanical alignment in TKA: a meta‐analysis and subgroup analysis of randomised trials. Arch Orthop Trauma Surg. 2018;138(9):1293–1303. 29961093 10.1007/s00402-018-2988-8

[33] Young SW , Walker ML , Bayan A , Briant‐Evans T , Pavlou P , Farrington B . The Chitranjan S. Ranawat Award: no difference in 2‐year functional outcomes using kinematic versus mechanical alignment in TKA: a randomized controlled clinical trial. Clin Orthop Relat Res. 2017;475(1):9–20. 27113595 10.1007/s11999-016-4844-x PMC5174030

